# *Senecio scandens* Buch.-Ham. polysaccharides exert anti-atopic dermatitis effects by modulating gut microbiota and the MAPK/NF-κB pathway

**DOI:** 10.3389/fphar.2025.1573135

**Published:** 2025-03-26

**Authors:** Zhi-Qin Hu, Shu-Shu Xie, Ming-Yuan Zhou, Yu-Chi Chen, Fang-Mei Zhou, Zhi-Shan Ding, Xiao-Qing Ye

**Affiliations:** School of Medical Technology and Information Engineering, Zhejiang Chinese Medical University, Hangzhou, Zhejiang, China

**Keywords:** *Senecio scandens* Buch.-Ham., polysaccharides, atopic dermatitis, anti-inflammatory, gut–skin axis

## Abstract

This study aims to extract polysaccharides from *Senecio scandens* Buch.-Ham. (SSP) using alcohol and water extraction and investigate whether they can be delivered orally to treat atopic dermatitis (AD). *In vivo* investigations demonstrated that SSP notably improved inflammation in mice, reducing ear swelling, scratching frequency, mast cell infiltration, and epidermal thickness. Furthermore, it lowered the levels of associated inflammatory markers, increased the production of skin barrier-associated proteins, and restored gut microbial diversity, which altered the composition of bacterial communities. *In vitro* experiments demonstrated that SSP could diminish the levels of inflammatory factors in the human immortal keratinocyte line (HaCaT) and suppress the MAPK/NF-κB signaling pathway. Our results suggest SSP exerts anti-AD effects and regulates the gut–skin axis in mice. The anti-inflammatory mechanism involves the MAPK/NF-κB signaling pathway. It is being tested for development into an effective drug for AD.

## 1 Introduction

Atopic dermatitis (AD) is a recurrent, chronic, non-infectious inflammatory skin disease characterized by typical pruritic eczematous skin lesions with epidermal barrier damage and skin immunological changes (Balakirski and Novak, 2022). The prevalence of AD ranges from 15% to 20% in children and up to 10% in adults (Schuler et al., 2023). AD can occur at any age and imposes a substantial life burden on patients (Ben Abdallah and Vestergaard, 2022). In a subset of patients, AD precedes other atopic diseases and progresses to food allergy, asthma, and allergic rhinitis (Fishbein et al., 2020). This type of skin inflammation is also a trigger for diseases such as endometriosis, liver dysfunction, and cancer (Itamura and Sawada, 2022).

Nowadays, topical corticosteroids are the first-line treatment option for AD. However, in some patients, steroid therapy is difficult to achieve (Wang et al., 2021). Dupilumab is a non-steroidal treatment approved by the Food and Drug Administration. Unfortunately, it is expensive, and antagonism of the Th2 pathway with dupilumab may lead to the activation of the Th1 pathway in patients, leading to Th1-mediated diseases such as psoriasis (Fowler et al., 2019).

Traditional Chinese medicine (TCM) has a long history of treating inflammatory diseases. In the past few years, numerous research studies have proven that certain TCMs and their key elements possess significant anti-inflammatory properties (Yan et al., 2020; Song et al., 2021; Yang et al., 2021). *Senecio scandens* Buch.-Ham. (SS) is an Asteraceae plant with a long history of medicinal use and is widely distributed throughout the world. According to the records in *Supplement to Materia Medica*, SS and its prescriptions have the effects of eliminating heat and exhibiting antibacterial properties. In TCM, SS is mainly used to treat various diseases such as abscess furuncle, bacillary dysentery, eczema, gonorrhea, and heat block. SS has been included in the 2020 edition of the Chinese Pharmacopoeia, which records that SS cleans away heat and detoxicates, brightens the eyes, and reduces dampness. It is used for carbines, swollen sores, cold and fever, red and swollen eyes, diarrhea and dysentery, and skin eczema. Studies have shown that SS extract has broad-spectrum antibacterial activity against *Staphylococcus aureus* (Wang et al., 2013). The total flavonoids of SS have been shown to inhibit auricular swelling and granuloma proliferation. It suppressed the production and release of the inflammatory factors (Wang et al., 2013). The water extract of SS could reduce the number of mast cells in mouse skin and improve pruritus symptoms by inhibiting the activity of MrgprB2 receptors (Ye et al., 2022). Compounds such as natural polysaccharides are widely used in the biomedical field to treat AD due to their therapeutic effects and relatively low toxicity. SS contains approximately 6.85% polysaccharide content (Dou et al., 2017), which shows a potential immune stimulatory effect (Yu et al., 2018). Therefore, given the remarkable anti-inflammatory and antibacterial properties of SS and its traditional role in treating eczema, we hypothesized that *Senecio scandens* Buch.-Ham. polysaccharides (SSP), as one of the main components of SS, might also play a role in treating AD.

Investigating the pharmacological effects and mechanisms of SSP is crucial given its enormous therapeutic potential for AD. In order to generate new insights into the use of TCM polysaccharides in the treatment of atopic disorders, this research explored the impact of SSP on both *in vivo* and *in vitro* AD models.

## 2 Materials and methods

### 2.1 Chemicals and reagents

Calcipotriol (MC903) was purchased from MedChemExpress (NJ, United States). The dexamethasone cream was purchased from Henan Daxin Pharmaceutical Industry Co., Ltd. (Henan, China). Filaggrin (FLG) antibody was obtained from Santa Cruz Biotechnology, Inc. (sc-66192, CA, United States). Antibodies for p-ERK (9101), ERK (9102), p-JNK (9251), JNK (9252), p-p38 (9211), and p38 (9212) were purchased from Cell Signaling Technology, Inc. (Danvers, MA, United States). Antibodies for p-p65 (YP0191) and p65 (YM3111) were purchased from ImmunoWay (Texas, United States). Proteintech (Chicago, United States) provided the HRP-conjugated AffiniPure Goat Anti-Mouse IgG (SA00001-1), HRP-conjugated AffiniPure Goat Anti-Rabbit IgG (SA00001-2), loricrin (LOR, 55439-1-AP), anti-mouse β-actin (60008-1-Ig), and anti-mouse GAPDH (60004-1-Ig) antibodies. TNF-α (300-01A) and IFN-γ (300-02) were obtained from PeproTech (Cranbury, NJ, United States).

### 2.2 Preparation of SSP

The aboveground section of SS was purchased from Hangzhou Sanri Agri-Tech Co., Ltd. (Hangzhou, China) and identified by Prof. Ding Zhishan of Zhejiang Chinese Medical University. It was dried and ground to powder. The powder was heated twice with 95% ethanol in a 1:15 ratio (w/v) at 40°C for 2 h each, and the extract was filtered. The filter residue was combined. Then, it was added to 800 mL of pure water and heated at 70°C for 2 h, being centrifuged at 4,000 rpm. The supernatant was then collected. The precipitated part was combined, and the abovementioned steps were repeated. At 70°C and lower pressure, the mixed supernatant was collected and concentrated using a rotary evaporator. With continuous stirring, 95% ethanol was gradually added to the solution. The resulting precipitate was collected and dissolved in pure water, following overnight storage at 4°C and centrifugation. After removing ethanol by rotating and evaporating the solution at 60°C under lower pressure, the solution was vacuum freeze-dried to SSP. The polysaccharide content of SSP was determined to be 0.20 ± 0.01 mg/mL by the phenol-sulfuric acid method, and the standard curve is available in Supplementary Figure S1.

### 2.3 Animals and treatment

Shanghai Slac Laboratory Animal Co. Ltd. (Shanghai, China) provided female Balb/c mice (6 weeks, 22 ± 2 g), which were kept in the Laboratory Animal Center of Zhejiang Chinese Medical University; the mice maintained with sufficient drinking water and diet under the following controlled conditions: a 12-h light–dark cycle, 21°C–23°C temperature, and 45%–65% humidity. The research was conducted adhering to the European Union (EU) Directive 2010/63/EU on the protection of animals used for scientific purposes and was authorized by the Ethics Committee of Zhejiang Chinese Medical University (approval no.: IACUC-20231009-09).

**FIGURE 1:**
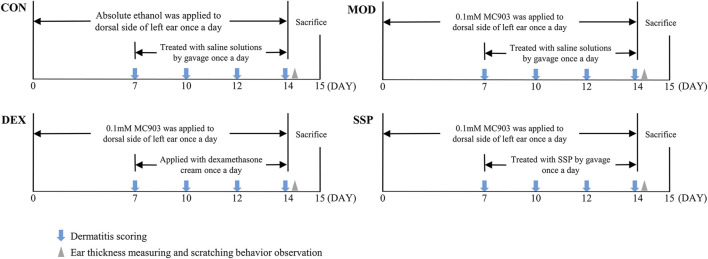
Outline of the methodology used in the animal experimentation.

Mice were randomly assigned to six groups, namely, CON, MOD, DEX, SSP-L, SSP-M, and SSP-H. The mouse model was set up on the basis of the method described by Hou et al. (2022). In brief, 2 nmol of MC903 dissolved in 20 μL absolute ethanol was applied to the left ear once daily for 14 days, except in the CON group, where an equal volume of absolute ethanol was applied. Starting from day 7, the CON and the MOD groups were given a saline solution by gavage, the DEX group was treated with 26 mg dexamethasone cream, and the mice in the SSP-treated group were given gavage treatments of 20 mg/kg (SSP-L), 50 mg/kg (SSP-M), and 100 mg/kg (SSP-H) SSP. The administration of drugs was performed in the morning, and MC903 or absolute ethanol was administered in the afternoon. On the last day, the mice to be sampled were placed in unpolluted cages. Fecal samples were taken right away following defecation, snap-frozen, and stored. After the mice were anesthetized, blood was collected through the retro-orbital venous plexus, and the mice were euthanized after head and neck dislocation. Blood samples were collected and centrifuged at 3,000 r/min for 10 min at 4°C, and serum was separated and then frozen in a refrigerator −80°C for further use. The spleens were bluntly isolated. The left ear was cut out and separated into two parts. One was soaked in 4% paraformaldehyde for fixation, while the other was frozen at −80°C. Figure 1 illustrates the animal experiment procedure.

### 2.4 Dermatitis scoring, ear thickness measuring, and scratching behavior observation

Dermatitis scores were performed on days 7, 10, 12, and 14. The dermatitis score includes four items, namely, erythema/bleeding, scales/dryness, edema, and exfoliation/erosion. The scores indicate no symptoms (0), mild symptoms (1), moderate symptoms (2), or severe symptoms (3), with a total score of 12 (Chen et al., 2020). After the last administration, the thickness difference (mm) between the left and right ears was calculated by measuring the thickness in the same areas of the two ears using a Vernier caliper. The number of scratching behaviors was recorded within 10 min.

### 2.5 Histopathological examination

The fixed left ears were embedded in paraffin for serial paraffin sectioning (5-μm-thick sections) and then stained with hematoxylin and eosin (HE) or toluidine blue. The pathological changes in the sections were observed under a microscope. A ×400 magnification was used to calculate the average amount of mast cells in five randomly selected fields.

### 2.6 Enzyme-linked immunosorbent assay

The levels of several inflammatory cytokines in blood were measured using enzyme-linked immunosorbent assay (ELISA) kits, including Immunoglobulin E (IgE, MM-0056M1), macrophage-derived chemokine (MDC/CCL22 and MM-0155M1), thymus activation-regulated chemokine (TARC/CCL17 and MM-0781M1), and regulated upon activation, normal T-cell expressed and secreted (RANTES/CCL5, MM-0903M1) (Jiangsu Meimian Industrial Co., Ltd., Jiangsu, China).

### 2.7 Gut microbiota analysis through 16S rRNA sequencing

Total DNA was isolated from mouse feces in accordance with the manufacturer’s recommendation. The bacterial 16S rRNA gene’s hypervariable region V3–V4 was amplified by adopting primers 338F (5′-ACT​CCT​ACG​GGA​GGC​AGC​AG-3′) and 806R (5′-GGACTACHVGGGTWTCTAAT-3′), using the DNA as a template (Liu et al., 2016). Polymerase chain reaction (PCR) products were extracted on 2% agarose gels, purified, and quantified using a Quantus™ Fluorometer (Promega, United States). According to the standard procedure of Majorbio Bio-Pharm Technology Co. Ltd. (Shanghai, China), sequencing was performed on an Illumina PE300 platform (Illumina, San Diego, United States). The raw read data were stored in the NCBI Sequence Read Archive (SRA) database. Quality control was carried out on the double-end original sequencing sequence using fastp (version 0.19.6) software (Chen et al., 2018). FLASH (version 1.2.11) software was utilized for merging (Magoč and Salzberg, 2011). Operational taxonomic units (OTUs) were clustered on the sequences after quality control and assembling according to 97% similarity, and chimera elimination was performed using UPARSE11 software (Edgar, 2013). To minimize the influence that sequencing depth brought to data analyses of alpha and beta diversity, sample sequence numbers were drawn flat. RDP Classifier (version 2.13) was used to compare the 16S rRNA gene database for OTU species taxonomic annotation with a confidence threshold of 70% (Wang et al., 2007). At different species taxonomic levels, each sample was statistically analyzed for the community composition.

### 2.8 Cell culture and treatment

The human immortal keratinocyte line (HaCaT) was cultured in DMEM medium (Gibco, Carlsbad, United States) supplemented with 10% fetal bovine serum (CellMax, Beijing, China) and 1% penicillin and streptomycin (Gibco, Carlsbad, United States). The culture conditions were 37°C and 5% CO_2_. When the confluence of cell growth reached 70%–80%, the cells were seeded in 96-well plates at a density of 1 × 10^4^ cells/well or in 6-well plates at a density of 5 × 10^5^ cells/well.

### 2.9 Cell viability

HaCaT cells were seeded in 96-well plates at a density of 1 × 10^4^ cells/well, and different concentrations of SSP (10, 30, 100, 300, and 1,000 μg/mL) were applied for 24 h. Following the manufacturer’s instructions, the Cell Counting Kit-8 (CCK-8, Biosharp, Beijing, China) was used to assess cell viability. CCK-8 solution was added to each well, and absorbance at 450 nm was measured using a spectrophotometer (BioTek Epoch 2, United States) after incubation for 1.5 h.

### 2.10 Real-time polymerase chain reaction

Total RNA was extracted from mouse left ear skin tissues and HaCaT cells using the FastPure Cell/Tissue Total RNA Isolation Kit V2 (RC112, Vazyme, Nanjing, China). The concentration of total RNA was measured at 260 nm using spectrophotometry. The contaminated DNA in the abovementioned samples was removed after being treated with the buffer solution. The BeyoRT^TM^ II cDNA Synthesis Kit with gDNA Eraser (D7170L, Beyotime, Shanghai, China) was used to generate first-strand cDNA with a total RNA content of 0.5–1.0 μg. The oligonucleotide primer sequences were manufactured by Bioengineering Co., Ltd. (Shanghai, China), as indicated in Supplementary Tables S1, S2. Real-time polymerase chain reaction (RT-PCR) was conducted in accordance with the manufacturer’s protocol using an ABI 7500 PCR system (A25742, Thermo Fisher Scientific, United States) and SYBR Green PCR Master Mix (Thermo Fisher Scientific, United States). The reference gene *GAPDH* was used to standardize the relative transcript levels of each gene, which were determined using the 2^−ΔΔCt^ method (Kim H. J. et al., 2022; Zeng et al., 2022).

### 2.11 Western blot

Mouse ear skin tissue samples were dissolved in the RIPA lysis buffer (Beyotime, Shanghai, China) with protease inhibitors (Beyotime, Shanghai, China), then homogenized using a hand-held homogenizer, and centrifuged. The supernatant was collected. After incubation with SSP for 4 h, HaCaT cells (5 × 10^5^ cells/well in 6-well plates) were treated with 10 ng/mL TNF-α/IFN-γ for 30 min and rinsed with ice-cold phosphate buffer (Biosharp, Beijing, China). The mixture was lysed with the RIPA lysis buffer containing protease inhibitors and centrifuged. The supernatant was collected and mixed in a 4:1 ratio with 5× loading buffer (Solarbio, Beijing, China). Protein concentrations were detected using the BCA Protein Assay Kit (Biosharp, Beijing, China). After electrophoresis on a 10% SDS polyacrylamide gel (Vazyme, Nanjing, China), proteins were transferred onto PVDF membranes (Millipore, United States) by electroblotting. The membranes were treated for 1 h at room temperature with the blocking solution (5% skim milk), followed by overnight incubation with primary antibodies at 4°C. Then, at room temperature, they were incubated for 2 h with secondary antibodies. The chemiluminescence imaging system (Tanon, Shanghai, China) and ECL reagent (Biosharp, Beijing, China) were utilized for visualization. Using ImageJ software, each band’s gray value was examined.

### 2.12 Statistical analysis

All quantitation data were presented as the mean ± SEM. A one-way ANOVA was used for the analysis of differences between the groups, and Tukey’s test was used for multiple comparisons. A *p*-value <0.05 was considered statistically significant.

## 3 Results

### 3.1 SSP alleviated the symptoms and pathology of MC903-induced AD in mice

**FIGURE 2:**
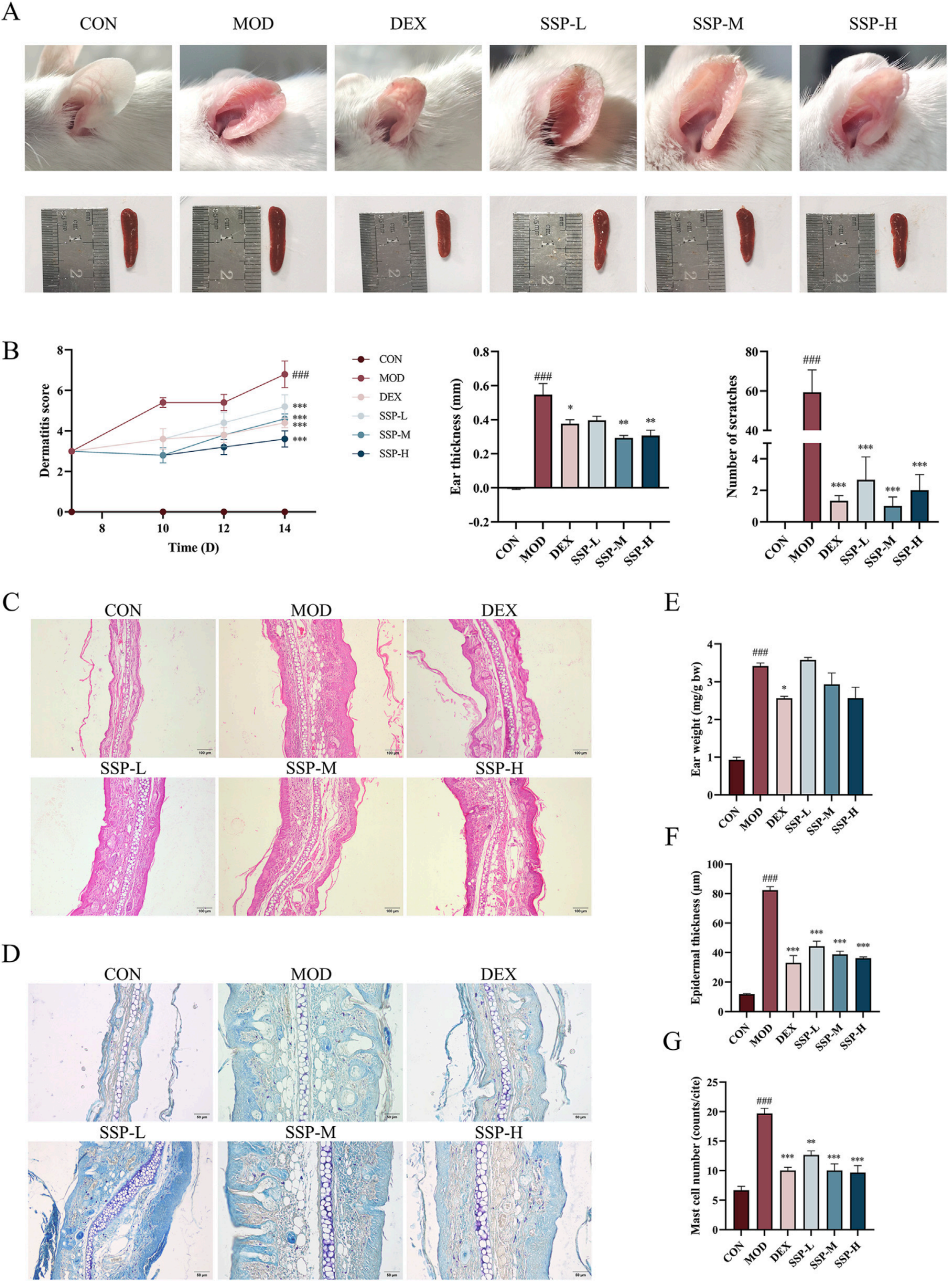
Impact of SSP on the symptoms and pathology in mice. **(A)** Ear and spleen changes. **(B)** Dermatitis severity scores (n = 5), the thickness differences between the left and right ears (n = 3), and the number of scratching behaviors (n = 3–5). **(C, D)** Ear histopathologic features. Epidermal thickening was assessed by HE staining (magnification, ×100), and mast cell infiltration was assessed by toluidine blue staining (magnification, ×200). **(E)** Ear weight changes (n = 3). **(F)** Epidermal thickness of HE-stained tissues (n = 6). **(G)** Expression of mast cells observed through toluidine blue-stained tissues (n = 3). MOD: MC903 (2 nmol/20 μL). SSP-L: SSP (20 mg/kg). SSP-M: SSP (50 mg/kg). SSP-H: SSP (100 mg/kg). Data were presented as the mean ± SEM. ^###^ *P* < 0.001 vs. CON group; ^*^ *p* < 0.05, ^**^ *p* < 0.01, and ^***^ *p* < 0.001 vs. MOD group.

The impact of SSP on AD symptoms in mice was evaluated through a series of assessments, including changes in ear lesions, spleen size, dermatitis scores, thickness differences between the left and right ears, and the number of scratches. Figure 2A shows that the MOD group’s ear skin was much redder and thicker than that of the CON group, with edema and surface injury. Dexamethasone and SSP administration alleviated redness and edema, although the latter resulted in a slight degree of dryness in the ear. At the same time, compared with those of the CON group, the spleens of the MOD group were obviously enlarged. The administration of dexamethasone and SSP, to a certain extent, alleviated splenomegaly. As illustrated in Figure 2B, each dosage of SSP decreased the AD dermatitis score and considerably decreased the mice’s scratching behavior compared to the MOD group (*p* < 0.001). SSP-M and SSP-H dramatically decreased the ear thicknesses (*p* < 0.01), despite the fact that the ear weights of the SSP treatment groups did not differ significantly from those of the MOD group (*p* > 0.05, Figure 2E). Ear skin lesions were further explored through pathological analysis. HE staining and epidermal thickness analysis showed that the CON group had an epidermal thickness of 11.82 ± 0.3576 μm, while the MOD group had an epidermal thickness of 82.29 ± 2.434 μm. The dexamethasone, SSP-L, SSP-M, and SSP-H groups had epidermal thicknesses of 32.93 ± 5.042 μm, 44.34 ± 3.341 μm, 38.76 ± 2.191 μm, and 36.08 ± 0.9661 μm, respectively. Three doses of SSP significantly reduced epidermal thickness compared to the MOD group *(p* < 0.001, Figures 2C, F). Toluidine blue staining and mast cell counting results showed that the MOD group expressed the most mast cells, while dexamethasone (*p* < 0.001) and SSP administration significantly reduced the expression of mast cells (*p* < 0.01 and *p* < 0.001) (Figures 2D, G). Dose-dependent effects were observed in the epidermal thickness and mast cell counts of the SSP-treated groups. These results indicated that SSP ameliorated AD and alleviated inflammatory responses in mice.

### 3.2 SSP improved serum inflammatory factor levels and repaired skin damage in AD mice

**FIGURE 3:**
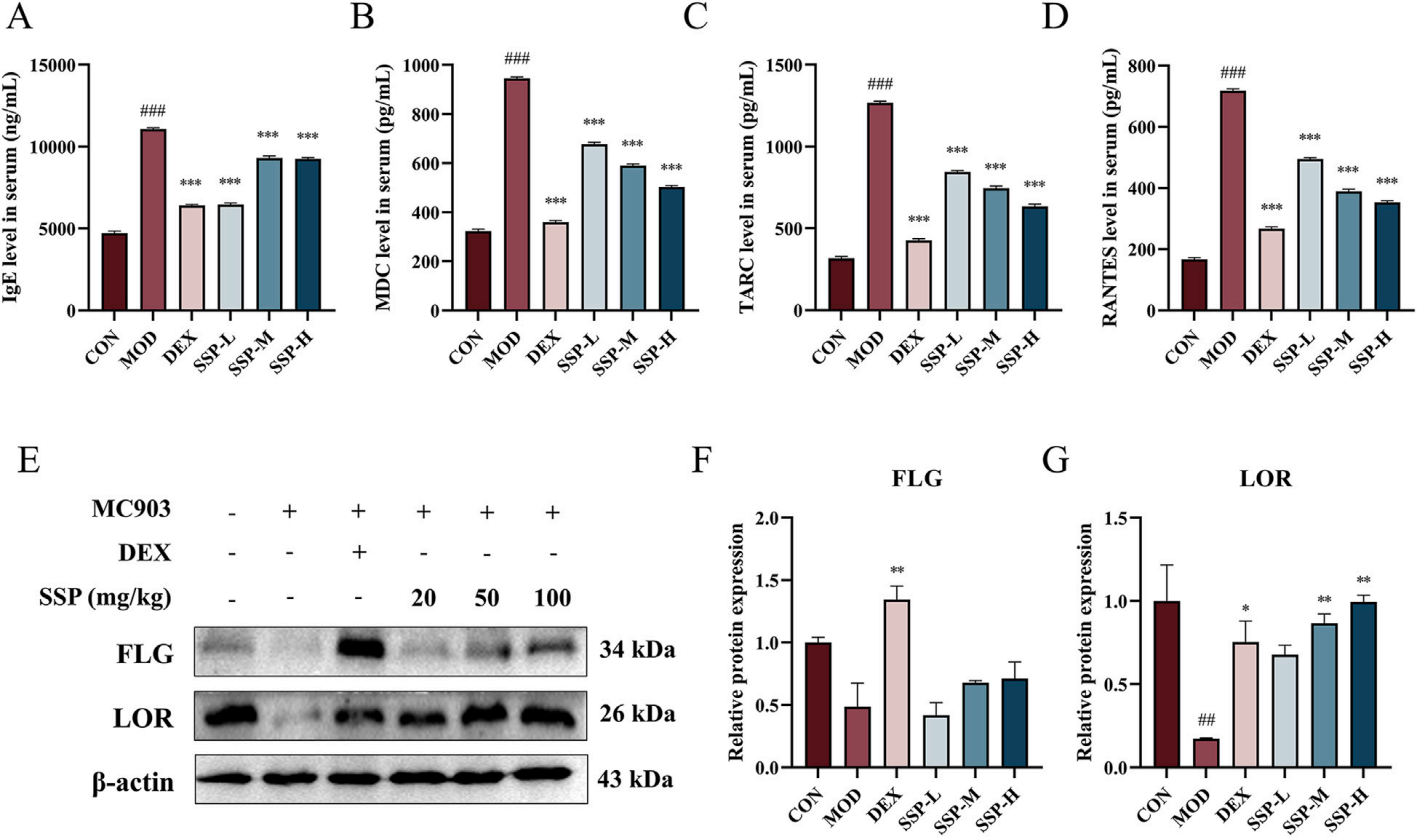
Impact of SSP on serum inflammatory factors and skin barrier protein expressions in mice. **(A–D)** IgE, MDC, TARC, and RANTES levels in the serum (n = 6). **(E–G)** FLG and LOR expression levels in the skin (n = 3). MOD: MC903 (2 nmol/20 μL). SSP-L: SSP (20 mg/kg). SSP-M: SSP (50 mg/kg). SSP-H: SSP (100 mg/kg). Data were presented as the mean ± SEM. ^##^ *P* < 0.01, ^###^ *p* < 0.001 vs. CON group; ^*^ *p* < 0.05, ^**^ *p* < 0.01, and ^***^ *p* < 0.001 vs. MOD group.

AD leads to Th1/Th2 cell dysfunction, and the overproduction of Th2 cells stimulates mast cell activation, increases IgE levels in the blood, and enhances the production of several chemokines. ELISA was used to detect the expression levels of related inflammatory factors in the blood. As shown in Figures 3A–D, the presence of MC903 significantly increased IgE, MDC, TARC, and RANTES expression levels in AD mice compared to those in the CON group (*p* < 0.001). The concentration of SSP determines its inhibitory action on MDC, TARC, and RANTES. However, the effect of dexamethasone was still superior to that of the three doses of SSP. The expression of the skin proteins FLG and LOR is significantly suppressed by the inflammatory cytokines generated by AD lesions, resulting in a destruction of the epidermal barrier. Figures 3E–G displays the findings of using Western blot to identify the expression of related proteins in the skin of AD mice. The MOD group showed lower expression of FLG and LOR protein than the CON group, and the LOR protein result was statistically different (*p* < 0.01). Although there was no significant difference in the expression of FLG protein, the medium- and high-dose SSP treatment significantly increased the expression of LOR (*p* < 0.01) compared to the MOD group. These findings showed that MC903 damaged the normal skin barrier, and SSP was able to restore partial integrity of the skin barrier by increasing the expression of LOR.

### 3.3 SSP inhibited mRNA expression induced by MC903

**FIGURE 4:**
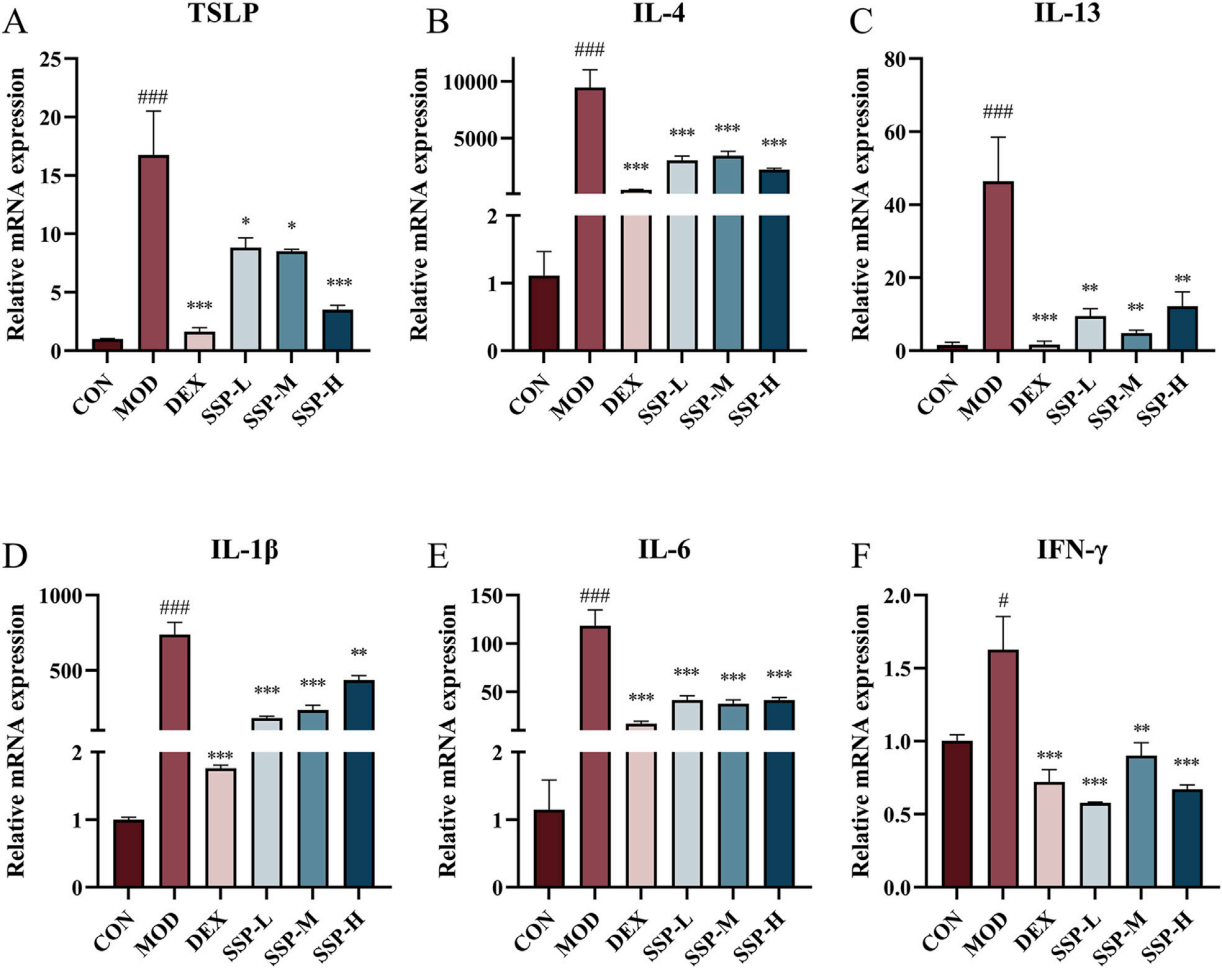
Impact of SSP on relative mRNA expressions in the ear. **(A–F)** Relative amounts of TSLP, IL-4, IL-13, IL-1β, IL-6, and IFN-γ mRNA expression (n = 3). MOD: MC903 (2 nmol/20 μL). SSP-L: SSP (20 mg/kg). SSP-M: SSP (50 mg/kg). SSP-H: SSP (100 mg/kg). Data were presented as the mean ± SEM. ^#^ *P* < 0.05, ^###^ *p* < 0.001 vs. CON group; ^*^ *p* < 0.05, ^**^ *p* < 0.01, and ^***^ *p* < 0.001 vs. MOD group.

Due to the predominance of Th2 cells, several inflammatory factors become important participants in the pathogenesis of AD. Alongside these inflammatory factors, Th2 cells infiltrate the skin, intensifying the inflammatory response. The mRNA expressions of TSLP, IL-4, IL-13, IL-1β, IL-6, and IFN-γ in the ear were determined using RT-qPCR. Figure 4 presents the findings. The mRNA levels of associated inflammatory factors in the MOD group were considerably higher than those in the CON group (*p* < 0.05 and *p* < 0.001). The expression of associated mRNA was significantly lower in the DEX and SSP groups than in the MOD group (*p* < 0.05, *p* < 0.01, and *p* < 0.001). The SSP group’s degree of reduction was still lower than that of DEX’s, with the exception of IFN-γ. These findings imply that SSP can successfully lower the level of Th2-related inflammatory cytokine mRNA expression, preventing the inflammatory response triggered by MC903.

### 3.4 SSP influenced gut microbial diversity in AD mice

**FIGURE 5:**
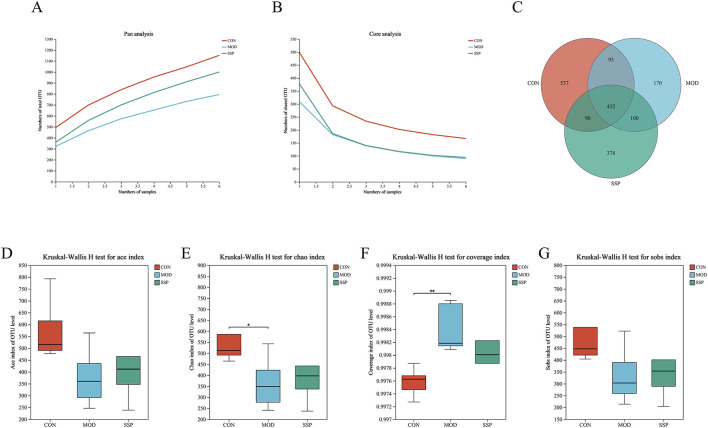
Gut microbial composition and alpha diversity in mice. **(A, B)** Pan/core curve of the samples (n = 6). **(C)** Venn diagram that showed OTU composition (n = 6). **(D–G)** Ace, Chao, coverage, and Sobs indices of OTU levels (n = 6). MOD: MC903 (2 nmol/20 μL). SSP: SSP (100 mg/kg). Mothur (version v.1.30.2) was used for the alpha diversity index analysis. The boot (version 1.3.18) and stats (version 3.3.1) packages in R language (version 3.3.1) were used for inter-index difference testing. ^*^ *P* < 0.05 and ^**^ *p* < 0.01 vs. CON group.

Gut microbiota changes caused by MC903 and the effect of SSP intervention on gut microbial diversity were investigated. After quality control, 1,071,256 high-quality 16S rRNA reads were acquired. A total of 7,258 bacterial OTUs were obtained through 97% OTU clustering. As shown in Figures 5A, B, with the increase in sample size, the number of pan species increased and then leveled off, while the number of core species decreased and then leveled off, which indicated that the sequencing samples were sufficient. A Venn diagram was employed to show sample composition similarity and the overlap of OTUs (Figure 5C). The three groups share a total of 432 OTUs. The CON group had the most unique OTUs, whereas the MOD group had the fewest. Figures 5D–G displays the alpha diversity index analysis. The MOD group had lower Ace, Chao (*p* < 0.05), and Sobs indices of gut microbiota than those of the CON group but had higher coverage indices (*p* < 0.01). The SSP group’s Ace, Chao, and Sobs showed increased indices compared to those of the MOD group, but the coverage index decreased, which showed that SSP can increase gut microbiota richness and reduce community coverage.

**FIGURE 6:**
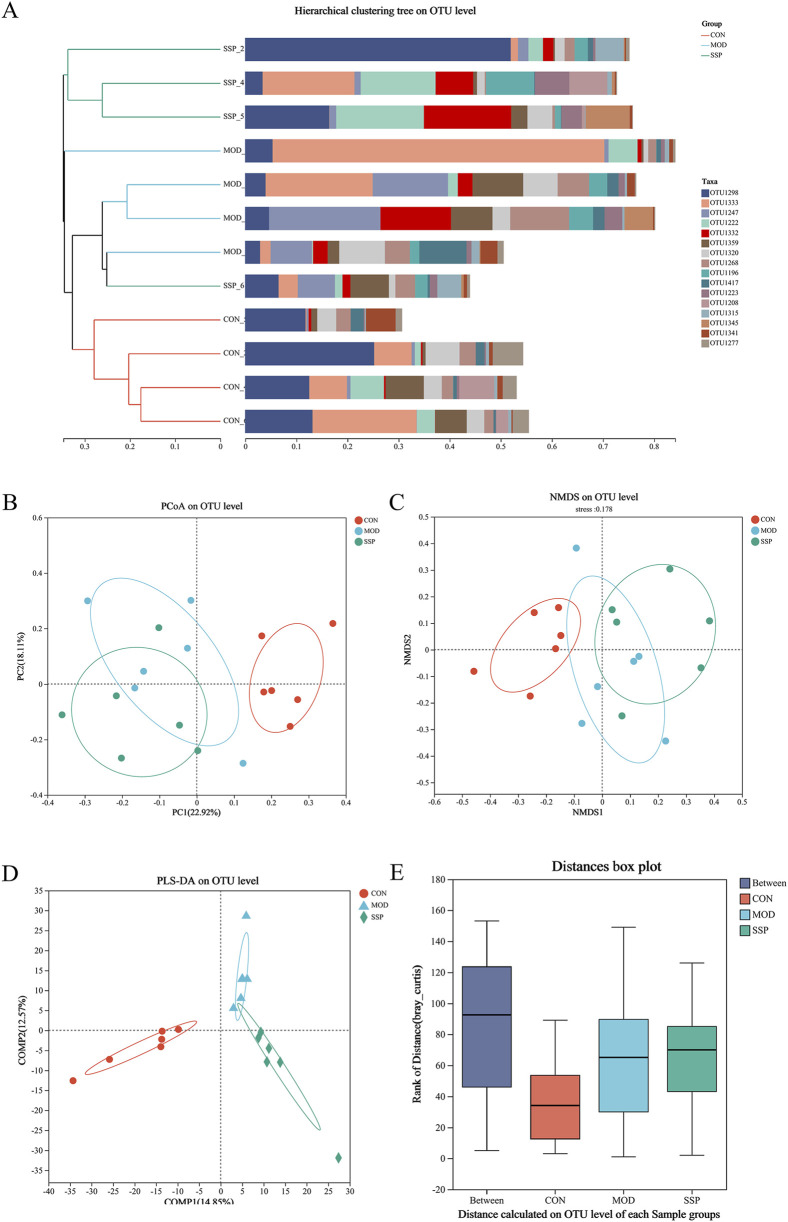
Gut microbial beta diversity in mice. **(A)** Hierarchical clustering results (n = 6). **(B–E)** PCoA, NMDS, PLS-DA, and ANOSIM analysis results (n = 6). The corresponding box represents the within-group difference distance, and the box of the between group represents the between-group difference distance. MOD: MC903 (2 nmol/20 μL). SSP: SSP (100 mg/kg). QIIME was used for calculating the beta diversity distance matrix, and then R language (version 3.3.1) was used for conducting statistical analysis and plotting.

The results of the beta diversity analysis are shown in Figure 6. According to the result of the hierarchical clustering tree, the disposal of the MOD group induced the change in gut microbial diversity in mice, and SSP can influence this change (Figure 6A). Grouping was reasonable since the ANOSIM box plot results showed that the median line in the between-group comparison was higher than that in the other groups (Figure 6E). According to the PCoA/NMDS/PLS-DA results, the CON group differed significantly from the MOD and SSP groups on the horizontal axis, whereas the MOD and SSP groups showed just a slight divergence. On the vertical axis, SSP was closer to the CON group compared to the MOD group. This suggested that in the horizontal direction, MC903 was the main impact factor, and in the vertical direction, SSP was the main impact factor (Figures 6B–D).

**FIGURE 7:**
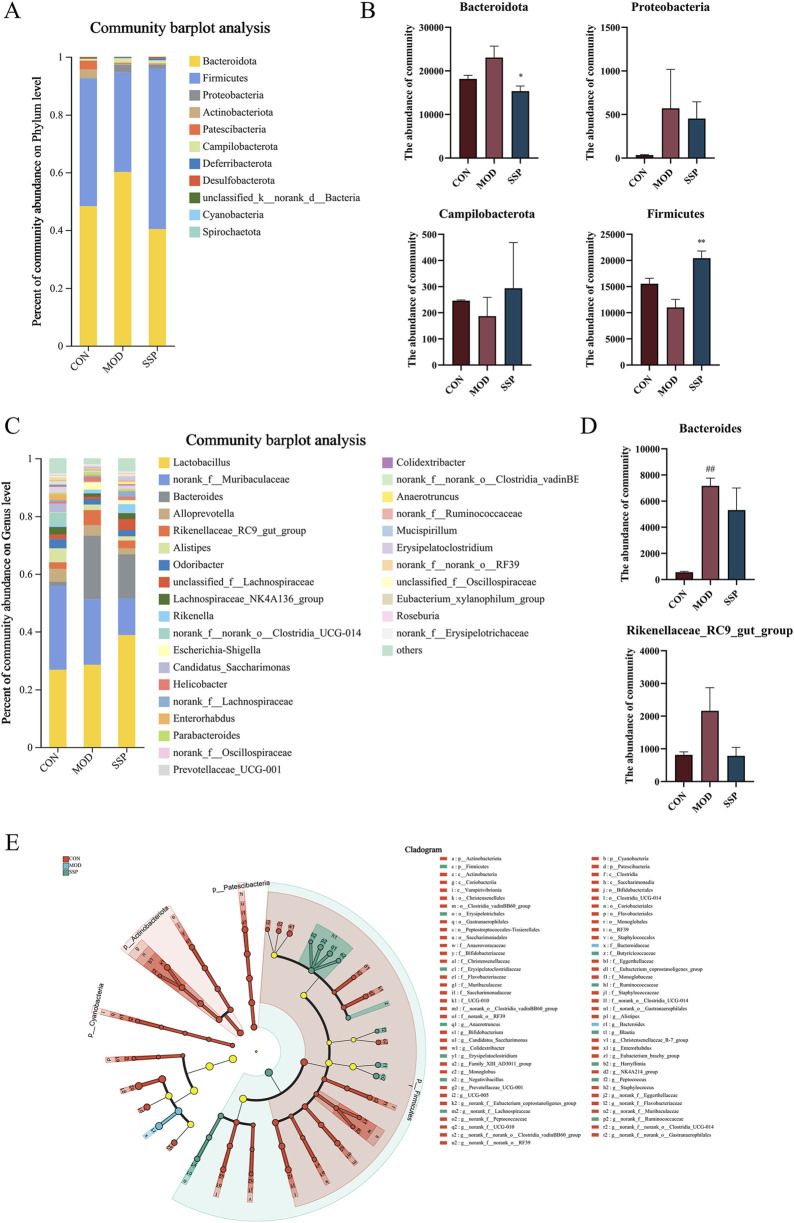
Gut microbial differences in community composition and species differences analysis in mice. **(A, B)** Relative microbial abundance in mice at the phylum level (n = 6). **(C, D)** Relative microbial abundance in mice at the genus level (n = 6). **(E)** LEfSe multi-level species discriminant analysis (n = 6). MOD: MC903 (2 nmol/20 μL). SSP: SSP (100 mg/kg). LEfSe software was used for LEfSe multi-level species discriminant analysis. ^##^ *p* < 0.01 vs. CON group; ^*^ *p* < 0.05; ^**^ *p* < 0.01 vs. MOD group.

Community composition results are shown in Figure 7. At the phylum level, six major bacterial phyla were identified, namely, *Bacteroidota*, *Actinobacteriota*, *Proteobacteria*, *Firmicutes*, *Patescibacteria*, and *Campylobacterota*. The MOD group had higher levels of *Bacteroidota* and *Proteobacteria* than the CON group, while *Firmicutes* and *Campylobacterota* were lower. The SSP group showed a decrease in *Bacteroidota* (*p* < 0.05) and *Proteobacteria* and an increase in *Firmicutes* (*p* < 0.01) and *Campylobacterota* (Figures 7A, B). Six major bacterial genera were identified at the genus level, namely, *Lactobacillus*, *norank_f_Muribaculaceae*, *Bacteroides*, *Alloprevotella*, *Rikenellaceae_RC9_gut_group*, and *Alistipes*. *Bacteroides* and *Rikenellaceae_RC9_gut_group* in the MOD group were more rich than in the CON group (*p* < 0.01), and they were reduced in the SSP group compared with the MOD group (Figures 7C, D). Species difference analysis result is shown in Figure 7E. The LEfSe multilevel hierarchy species tree showed that the CON group was enriched in the vast majority of microbial groups. Nodes of *f_Muribaculaceae* and *g_norank_f_Muribaculaceae genera* in the CON group, *f_Bacteroidaceae* and *g_Bacteroides* in the MOD group, and *p_Firmicutes* in the SSP group were large, showing significant enrichment.

### 3.5 SSP suppressed mRNA expression induced by TNF-α/IFN-γ in HaCaT cells

**FIGURE 8:**
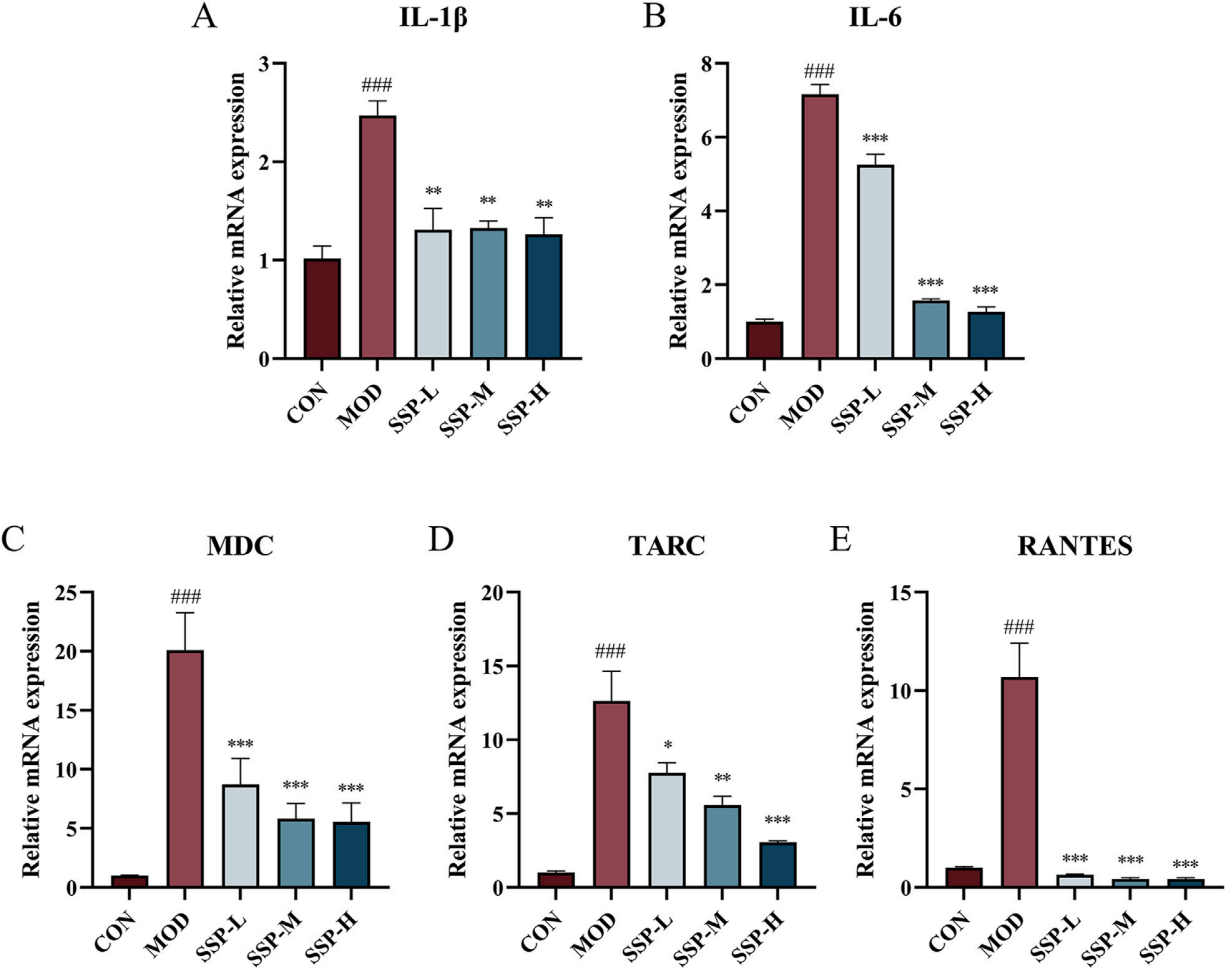
Impact of SSP on relative mRNA expressions in HaCaT cells. **(A-E)** Relative amounts of IL-1β, IL-6, MDC, TARC, and RANTES mRNA expression (n = 3). MOD: TNF-α/IFN-γ (10 ng/mL). SSP-L: SSP (6.25 μg/mL). SSP-M: SSP (25 μg/mL). SSP-H: SSP (100 μg/mL). Data were presented as the mean ± SEM. ^###^ *p* < 0.001 vs. CON group; ^*^ *p* < 0.05, ^**^ *p* < 0.01, and ^***^ *p* < 0.001 vs. MOD group.

As observed in Supplementary Figure S2, the impact of varying SSP doses on the proliferation activity of HaCaT cells was assessed using the CCK-8 assay. The concentration of SSP at 10 μg/mL did not significantly alter cell viability compared to the blank control group. At concentrations of 30, 100, 300, and 1,000 μg/mL, cell viability increased to 115.7%, 118.0%, 115.7%, and 112.6%, respectively (*p* < 0.05 and *p* < 0.01), suggesting that SSP had no effect on HaCaT cell proliferative activity. The potential of SSP in treating inflammation of HaCaT cells was evaluated by measuring the mRNA expression levels of cytokines IL-1β and IL-6 and chemokines MDC, TARC, and RANTES. HaCaT cells were seeded at a density of 5 × 10^5^ cells/well in 6-well plates. Cells were pretreated with varying SSP concentrations for 4 h before being treated with 10 ng/mL TNF-α/IFN-γ for 24 h. Cellular RNA was extracted for subsequent experiments. HaCaT cells can be stimulated by TNF-α and IFN-γ to produce associated inflammatory factors and chemokines. TNF-α/IFN-γ-stimulated HaCaT cells are frequently employed in *in vitro* models for AD research. As observed in Figure 8, RT-qPCR was utilized to measure the expression levels of the chemokines MDC, TARC, and RANTES mRNA, as well as the related cytokines IL-1β and IL-6. The mRNA expression of IL-1β, IL-6, TARC, MDC, and RANTES was considerably upregulated by TNF-α/IFN-γ compared to that in the CON group (*p* < 0.001), whereas SSP at 6.25, 25, and 100 μg/mL dosages significantly suppressed this expression (*p* < 0.05, *p* < 0.01, and *p* < 0.001). The abovementioned data imply that SSP has a moderating effect on the inflammatory response at the cellular level.

### 3.6 SSP inhibited the MAPK/NF-κB signal pathway in HaCaT cells stimulated by TNF-α/IFN-γ

**FIGURE 9:**
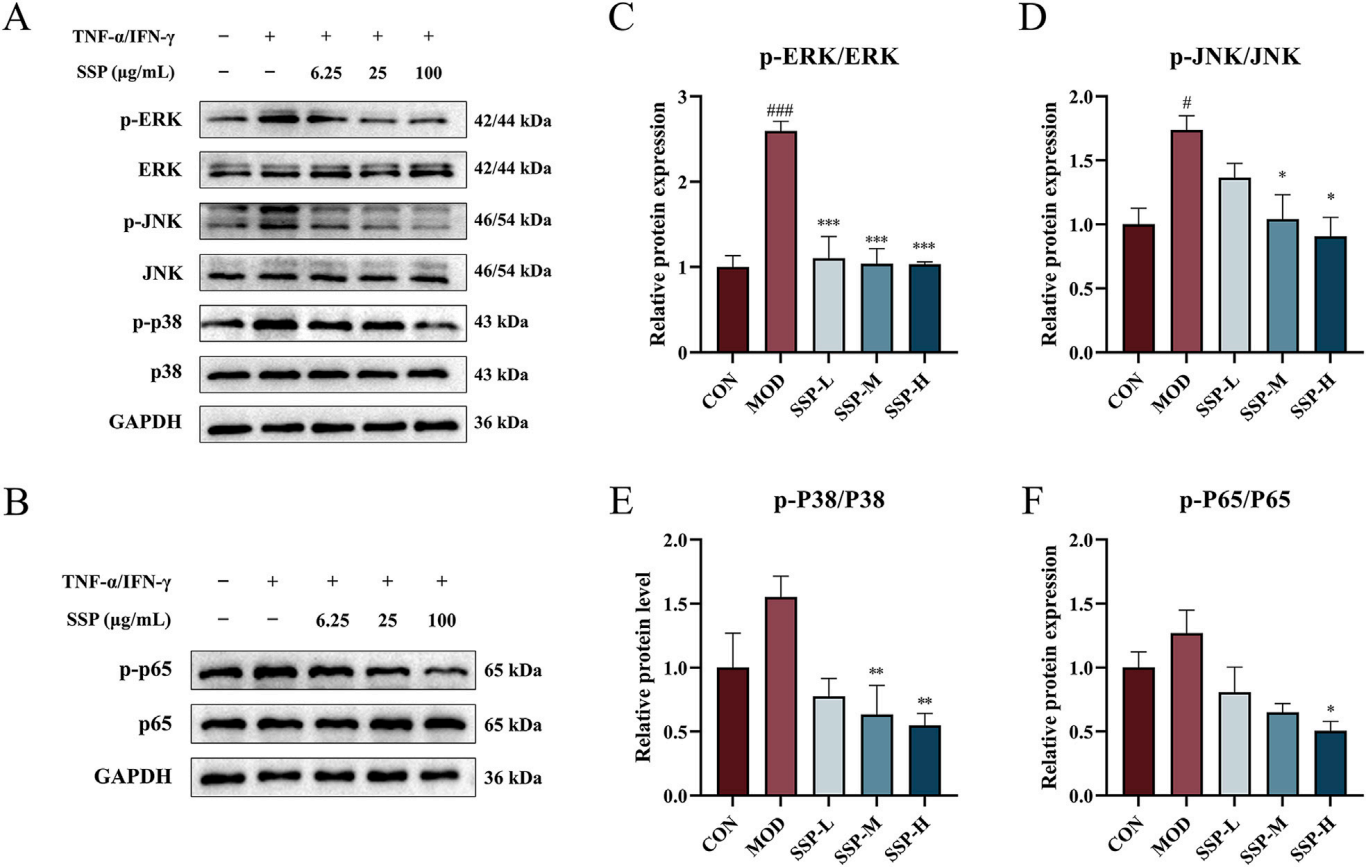
SSP’s effect on the MAPK/NF-κB pathway in HaCaT cells. **(A)** Expressions of the proteins p-ERK, p-JNK, p-p38, ERK, JNK, and p38 in HaCaT cells (n = 3). **(B)** Expression of the proteins p-p65 and p65 in HaCaT cells (n = 3). **(C–F)** Relative protein expression levels of p-ERK/ERK, p-JNK/JNK, p-p38/p38, and p-p65/p65 (n = 3). MOD: TNF-α/IFN-γ (10 ng/mL). SSP-L: SSP (6.25 μg/mL). SSP-M: SSP (25 μg/mL). SSP-H: SSP (100 μg/mL). Data were presented as the mean ± SEM. ^#^ *P* < 0.05, ^###^ *p* < 0.001 vs. CON group; ^*^ *p* < 0.05, ^**^ *p* < 0.01, ^***^ *p* < 0.001 vs. MOD group.

The inflammatory response increases pro-inflammatory cytokine levels and promotes MAPK protein phosphorylation, which initiates intracellular pathways. As shown in Figures 9A, C–E, the expression of three proteins linked to the MAPK pathways (ERK, JNK, and p38) in HaCaT cells was determined using Western blot. TNF-α/IFN-γ treatment in the MOD group resulted in higher production of phosphorylated ERK, JNK, and p38 proteins than that in the CON group. There was a considerable increase in p-ERK and p-JNK expressions (*p* < 0.05 and *p* < 0.001). SSP therapy reduced p-ERK, p-JNK, and p-p38 expressions in a dose-dependent way compared to the MOD group. All proteins showed a substantial decrease in expression at concentrations of 25 μg/mL (SSP-M) and 100 μg/mL (SSP-H) (*p* < 0.05, *p* < 0.01, and *p* < 0.001). According to these findings, SSP may reduce inflammation by blocking the MAPK signaling pathway.

One significant transcriptional regulator that is essential for inflammatory reactions is NF-κB. The expression of proteins linked to the NF-κB pathway in HaCaT cells was determined using Western blot, and the results are displayed in Figures 9B, F. TNF-α/IFN-γ treatment in the MOD group increased the expression of phosphorylated p65 protein compared to that in the CON group, while SSP treatment decreased the expression of phosphorylated p65 compared to that in the MOD group, and it was dose-dependent. At 100 μg/mL (SSP-H), the impact was significantly reduced (*p* < 0.05). These results suggested that SSP could alleviate inflammation by inhibiting the NF-κB signaling pathway.

## 4 Discussion

The mice in our study treated with MC903 alone exhibited severe redness and swelling, high dermatitis scores, and ear thickening, which were in line with previously published findings (Scibiorek et al., 2023; Jiang et al., 2023; Lu et al., 2024). This acute AD model successfully activated the Th2 immune response (Lu et al., 2024). SS has been proved to alleviate itching triggered by allergic contact dermatitis (Ye et al., 2022), and its polysaccharide component has demonstrated a low-toxicity immunomodulatory effect (Yu et al., 2018). This study is the first to examine the role and mechanism of SSP in the treatment of AD.

Destruction of the skin barrier, Th2 response changing, and itching are three main pathological factors of AD. The skin barrier protects against various injuries by forming keratinocytes (Yang et al., 2020). It can trigger the release of inflammatory cytokines (such as TSLP) and stimulate Th2 cells to secrete Th2 cytokines (such as IL-4 and IL-13) following the destruction of the epidermal barrier (Lu et al., 2024). These cytokines cause the occurrence of pruritus, and scratching caused by pruritus may exacerbate inflammation by accelerating lesion skin damage (Ye et al., 2022). IL-4 and IL-13 cause an increase in serum IgE levels and the infiltration of mast cells and eosinophils into the skin (Choi et al., 2021). Increased inflammatory cell infiltration into skin lesions leads to tissue injury, edema, and thickening via the production of inflammatory mediators. Consequently, pro-inflammatory cytokine overexpression, mast cell infiltration, thicker epidermis, and increased IgE release are typical characteristics of AD (Wang et al., 2022). Our study’s findings demonstrated that SSP can improve acute AD lesions induced by MC903, reduce dermatitis scores and scratches, and lighten ear swelling degree in mice. The spleen plays an important role in immunomodulation. Our study proved that SSP was able to alleviate splenomegaly. Similarly, a study showed that the ethanol extract of *Artemisia anomala* S. Moore (EAA) decreased the epidermis thickness and the size of the spleen in AD mice induced by 2,4-dinitrochlorobenzene (DNCB) (Yang et al., 2021). Our pathological investigation revealed that SSP dramatically decreased both the degree of inflammatory cell infiltration and the thickness of the epidermis. It caused the tissue’s mast cell count to decrease. The ethanol extract of *Artemisia apiacea* Hance (EAH) was also found to prevent epidermal thickening induced by DNCB in a dose-dependent manner (Yang et al., 2018). The inflammatory factors in mouse serum and ear tissue were examined in relation to the findings of previous experiments. IgE levels in the serum of mice treated with SSP were considerably lower, according to ELISA analysis. In addition, the results of RT-PCR showed a notable reduction in TSLP, IL-4, IL-13, IL-1β, IL-6, and IFN-γ cytokine mRNA expressions in the ear tissues of SSP-treated groups. Animal studies using *Tremella fuciformis* polysaccharides and fucoidan to treat AD in mice showed comparable outcomes (Chen et al., 2021; Xie et al., 2022). The abovementioned results suggested that SSP may help alleviate AD symptoms and lessen inflammation reactions.

Since SSP had a substantial impact on serum IgE secretion, the levels of some chemokines were also examined accordingly. Important indicators of the intensity of AD-like skin symptoms include TARC and MDC (Choi et al., 2021). TARC and MDC are expressed in early Th2 cell response, promoting Th2 cell recruitment to lesions with inflammation, and are markedly elevated in AD patients’ serum (Kim et al., 2024). Their levels correlate with the severity of AD (Jahnz-Rozyk et al., 2005; Frusciante et al., 2024). RANTES, a key inflammatory chemokine, can coordinate white blood cells at inflammation sites and acts as a chemotactic agent for T-cell activation (Frusciante et al., 2024). Keratinocytes, which are encased in a protein shell, differentiate to produce the skin barrier. The protein shell contains FLG and LOR. Epidermal barrier dysfunction results from the substantial inhibition of FLG and LOR mRNA and protein expression by IL-4 and IL-13, which are generated by AD (Furue et al., 2017). As a consequence, FLG and LOR expressions were also detected. Our findings demonstrated that SSP helped lower inflammation and repair the epidermal barrier by restoring serum levels of inflammatory chemokines (MDC, TARC, and RANTES) and FLG and LOR protein expression. Hou et al. (2022) showed that the AD mice exhibited restored TARC and FLG expression in the skin under corresponding drug treatment, which was in line with the abovementioned results.

To help clarify the significance of the gut–skin axis in the therapy of AD, the alterations in the gut microbiota were assessed in order to better explore the changes SSP caused in mice. A study has shown that a cesarean section and prenatal antibiotic exposure can reduce the diversity of infant fecal microbiota, thereby promoting the development of infantile AD (Lee et al., 2014). According to our research, SSP increased the gut microbiota’s community diversity in AD mice, suggesting that changes in diversity affect the development of atopic illnesses. The gut microbiota is mainly divided into *Bacteroidota* and *Firmicutes* (Kim D.-Y. et al., 2022). *Bacteroidota* can recognize, transport, and degrade simple carbohydrates and help break down complex carbohydrates (Wardman et al., 2022). *Firmicutes* can regulate the generation of intestinal short-chain fatty acids (SCFAs) (Tong et al., 2023). Fructo-oligosaccharides have been shown to reduce the proportion of intestinal *Bacteroidota* in AD mice while increasing the abundance of *Lactobacillus* (a member of *Firmicutes*) (Chen et al., 2023). A research study showed that maintaining proper gut homeostasis was linked to an increase in the *Firmicutes*/*Bacteroidota* (F/B) ratio (Dei-Cas et al., 2020). In our study, the MOD group mice had an increasing trend in the number of intestinal *Bacteroidota*, while the *Firmicutes* decreased. The SSP group exhibited a notable reduction in *Bacteroidota* and a considerable increase in *Firmicutes* compared to the MOD group. This resulted in an elevated F/B ratio, suggesting that the gut microbial homeostasis was changed. In addition, the increase in *Bacteroides* abundance is associated with type-2 immunity (Kim D.-Y. et al., 2022). A research study proved that the gut microbiota of AD patients was disrupted, with an increase in *Bacteroides* content (Mahdavinia et al., 2019). In our study, the MOD group’s *Bacteroides* levels were noticeably greater than those of the CON group, and SSP could reverse this trend. These findings showed that SSP influences the development of AD by changing the gut microbiota’s composition, and the mechanism may be related to the production of SCFAs.

After gaining some understanding of the *in vivo* changes, the mechanism of SSP was also explored. HaCaT cells activated by TNF-α/IFN-γ are frequently used *in vitro* models for AD studies (Gil et al., 2021; Shiu et al., 2022). TNF-α and IFN-γ can promote the expression of chemokines and inflammatory cytokines in keratinocytes (Takuathung et al., 2021; Shiu et al., 2022). IL-1β and IL-6 are important participants in the process of cytokine-mediated inflammation cell migration, keratinocyte proliferation, and further cytokine production (Frusciante et al., 2024). The study’s findings show that SSP can lower the expression of IL-1β, IL-6, MDC, TARC, and RANTES mRNAs in HaCaT cells stimulated by TNF-α/IFN-γ. EAA was also found to significantly reduce TARC, RANTES, and IL-6 levels in HaCaT cells treated with TNF-α/IFN-γ (Yang et al., 2021), which aligned with our findings. To learn more about the mechanism, the protein expressions of relevant inflammatory signaling pathways were detected. One important mechanism that is continuously involved in the regulation of inflammation is the MAPK/NF-κB signaling pathway. MAPK is mainly divided into three categories: mitogen-responsive ERK, stress-responsive JNK/SAPK, and p38 MAPK. The MAPK protein is phosphorylated in response to inflammation. Additionally, it speeds up the production of pro-inflammatory cytokines and the activation of intracellular pathways (Park et al., 2020). As a significant transcriptional regulator, NF-κB regulates TARC activity in AD-related skin inflammation and is essential for inflammatory responses (Choi et al., 2021). The joint regulation of the MAPK and NF-κB pathways is considered a treatment strategy for inflammatory diseases (Takuathung et al., 2021). Mast cells produce FcεR1, which binds to IgE to activate the MAPK and NF-κB signaling pathways. This causes mast cells to degranulate and release inflammatory mediators such as histamine and IL-4 (Kim et al., 2024). The phosphorylation of NF-κB and MAPK (including ERK1/2, JNK, and p38) was shown to be inhibited by SSP, indicating that SSP works through the MAPK/NF-κB pathway to reduce inflammation in HaCaT cells. According to studies conducted on Asteraceae plants, EAA decreased the increase in phosphorylated ERK and inhibited the expression of NF-κB in HaCaT cells (Yang et al., 2021). Moreover, EAH inhibited p38/ERK activation and p65 transfer to the nucleus (Yang et al., 2018). It appears to be a similar mechanism to our findings.

According to Jiang et al., *Poria cocos* extract regulated the expression of phosphorylated ERK1/2 and p38 MAPK and increased the number of beneficial bacteria in the gut of mice. This increased the diversity of the gut microbiota, with *Bacteroidetes* and *Rikenellaceae* having a significant correlation with p38-MAPK expression (Jiang and Fan, 2021). Lu et al. (2020) showed that phycocyanin might prevent intestinal damage in mice by controlling gut microbiota and preventing the activation of the TLR4/Myd88/NF-κB pathway (Lu et al., 2020). The gut microbiome influences the immune system and a network of molecular connections between them (Anders et al., 2013). The resident microbes support healthy immune system development and control the signals of the immunological response that follows (Gao et al., 2018). Thus, it is easy to conclude that gut microbiota and inflammatory molecular signaling are inextricably linked, and more research is needed on this relationship.

The three limitations to this study are as follows: (1) this study explained the mechanism of SSP in HaCaT cells, but the mechanism in mice remains to be elucidated. (2) This study tested the AD mice spleen size, left and right ear thickness difference, scratching behaviors, and other symptoms. However, it is necessary to explore in-depth the epidermal barrier and other physical indicators, such as skin moisture loss, etc. (3) This study adopted the crude polysaccharides of SS as the research object. Nonetheless, crude polysaccharides are complex in composition and rich in impurities. The substances that play a role and their exact amounts remain a mystery, requiring further exploration in future research.

## 5 Conclusion

The *in vivo* experiment demonstrated that SSP improved AD symptoms in MC903-induced mice by repairing the damaged epidermal barrier and regulating inflammatory factor levels. By controlling the gut microbial diversity and the F/B ratio, it restored gut homeostasis. According to *in vitro* experiments, by blocking the MAPK/NF-κB signaling pathways, SSP was able to control inflammation in HaCaT cells that TNF-α and IFN-γ caused. It can, therefore, be concluded that SSP intervention exerts anti-inflammatory effects through immune regulation.

## Data Availability

The data presented in the study are deposited in the NCBI Sequence Read Archive (SRA) database (Accession Number: PRJNA1237259).
