# Supplementary figures and images for: Senecio scandens Buch.-Ham. polysaccharides exert anti-atopic dermatitis effects by modulating gut microbiota and the MAPK/NF-κB pathway
Source: Front Pharmacol. 2025 Mar 26;16:1573135. doi: 10.3389/fphar.2025.1573135 (PMC11978834; doi:10.3389/fphar.2025.1573135)

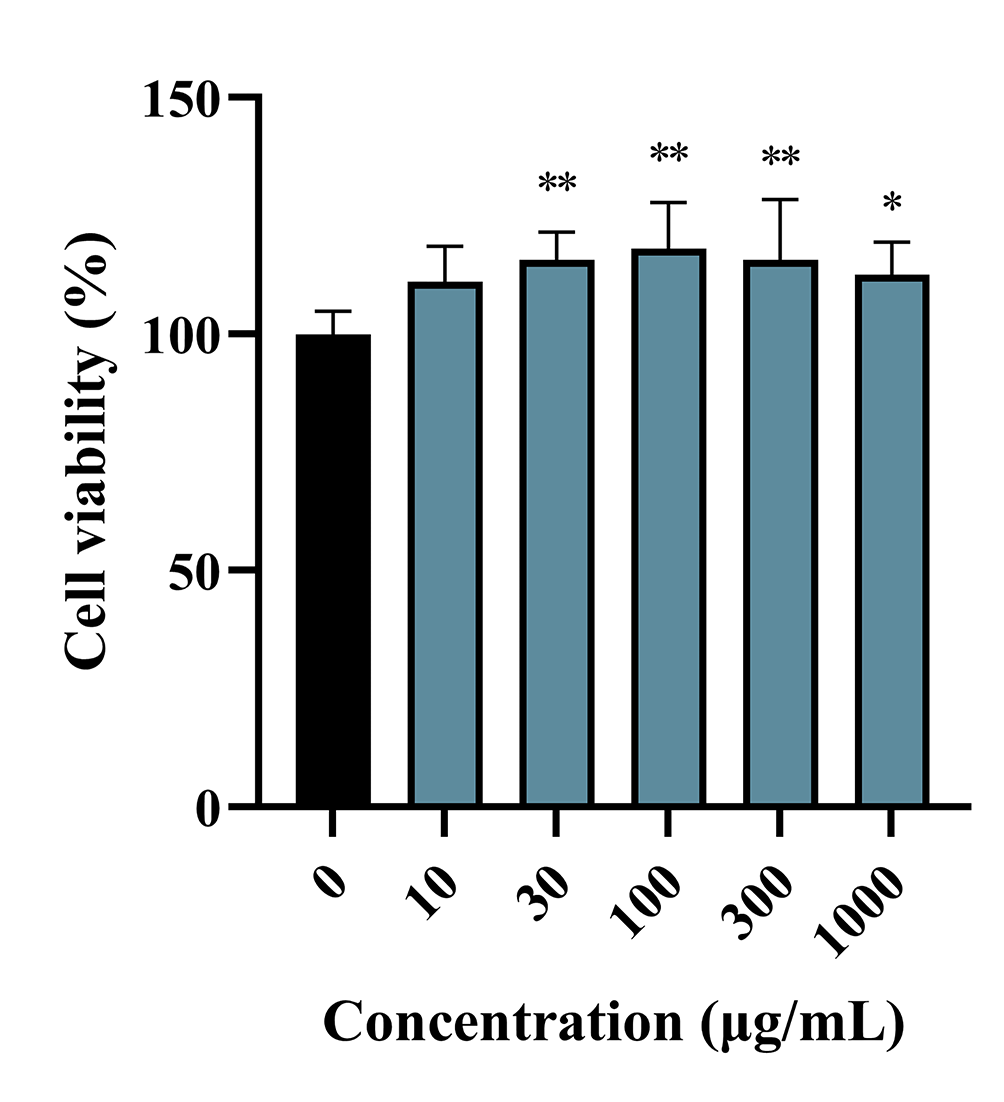

Supplement: Supplementary file 1 [file Image2.tif]

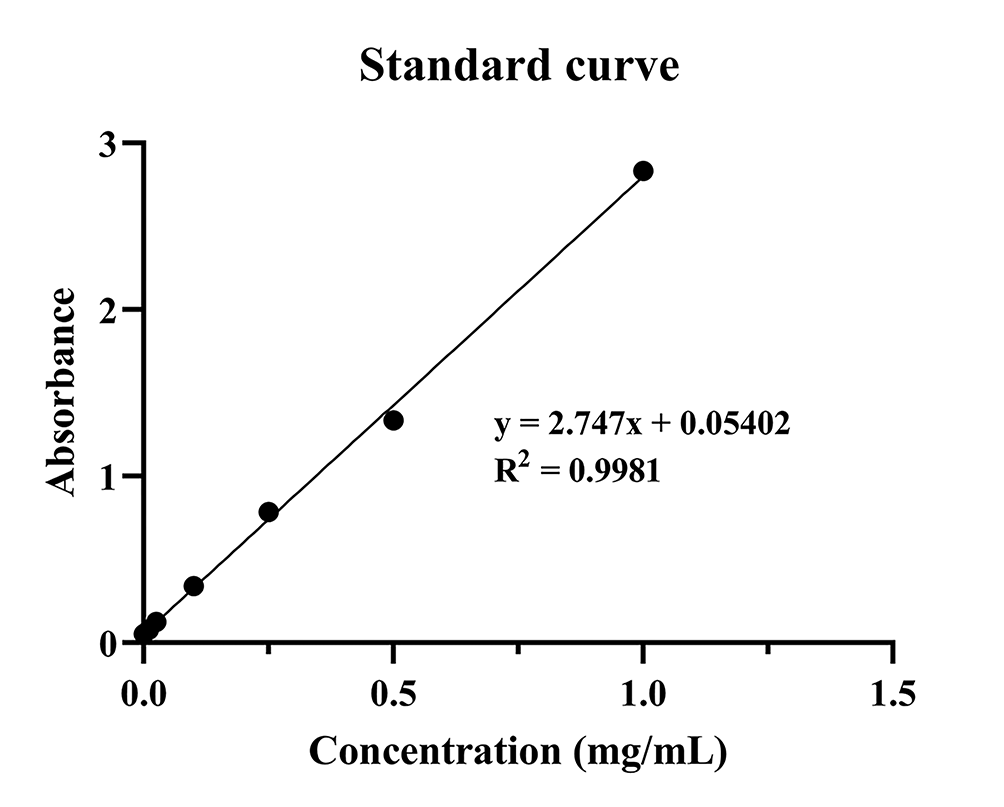

Supplement: Supplementary file 2 [file Image1.tif]
