# Supplementary material for: Senecio scandens Buch.-Ham. polysaccharides exert anti-atopic dermatitis effects by modulating gut microbiota and the MAPK/NF-κB pathway
Source: Front Pharmacol. 2025 Mar 26;16:1573135. doi: 10.3389/fphar.2025.1573135 (PMC11978834; doi:10.3389/fphar.2025.1573135)
Supplement: Supplementary file 3 [file DataSheet1.docx]

Supplementary Material

**Table S1** Mice RT-qPCR primer sequence.

| Gene | Forward primer (5′–3′) | Reverse primer (5′–3′) |
| --- | --- | --- |
| *TSLP* | AGCTTGTCTCCTGAAAATCGAG | AGGTTTGATTCAGGCAGATGTT |
| *IL-4* | TACCAGGAGCCATATCCACGGATG | TGTGGTGTTCTTCGTTGCTGTGAG |
| *IL-13* | TGAGGAGCTGAGCAACATCA | ATTTTGGTATCGGGGAGGCT |
| *IL-1β* | TGGACCTTCCAGGATGAGGACA | GTTCATCTCGGAGCCTGTAGTG |
| *IL-6* | AGTTGCCTTCTTGGGACTGA | TCCACGATTTCCCAGAGAAC |
| *IFN-γ* | GAGCCTAGAGACTATCACACCG | TACCAGAGGGTGTAGTTAGCGG |
| *GAPDH* | ATGGTGAAGGTCGGTGTGAA | TGGAAGATGGTGATGGGCTT |

**Table S2** Human RT-qPCR primer sequence.

| Gene | Forward primer (5′–3′) | Reverse primer (5′–3′) |
| --- | --- | --- |
| *IL-1β* | CTCTCACCTCTCCTACTCACT | ATCAGAATGTGGGAGCGAAT |
| *IL-6* | CGAGCCCACCGGGAACGAAA | GGACCGAAGGCGCTTGTGGAG |
| *MDC* | GTTGTCCTCGTCCTCCTTGC | GGAGTCTGAGGTCCAGTAGAAGTG |
| *TARC* | GTCTTGAAGCCTCCTCACCC | GGATCTCCCTCACTGTGGCT |
| *RANTES* | CGCTGTCATCCTCATTGCTA | GCACTTGCCACTGGTGTAGA |
| *GAPDH* | GGAGCGAGATCCCTCCAAAAT | GGCTGTTGTCATACTTCTCATGG |


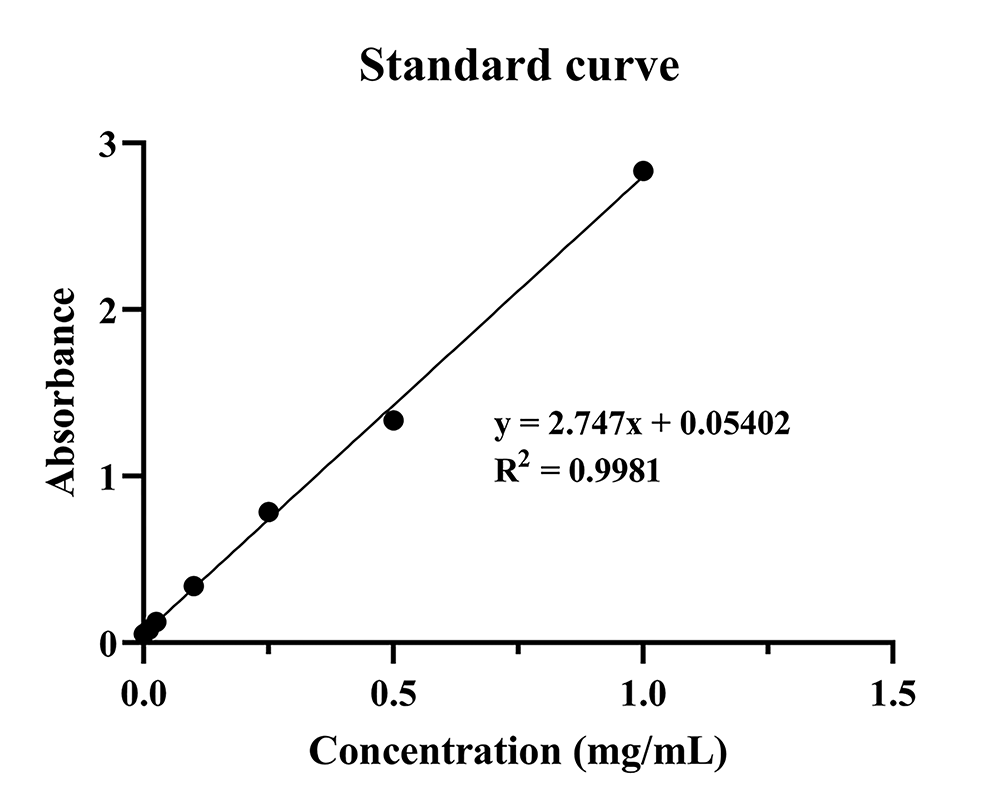


**Fig. S1.** Standard curve of polysaccharide content detection.

**
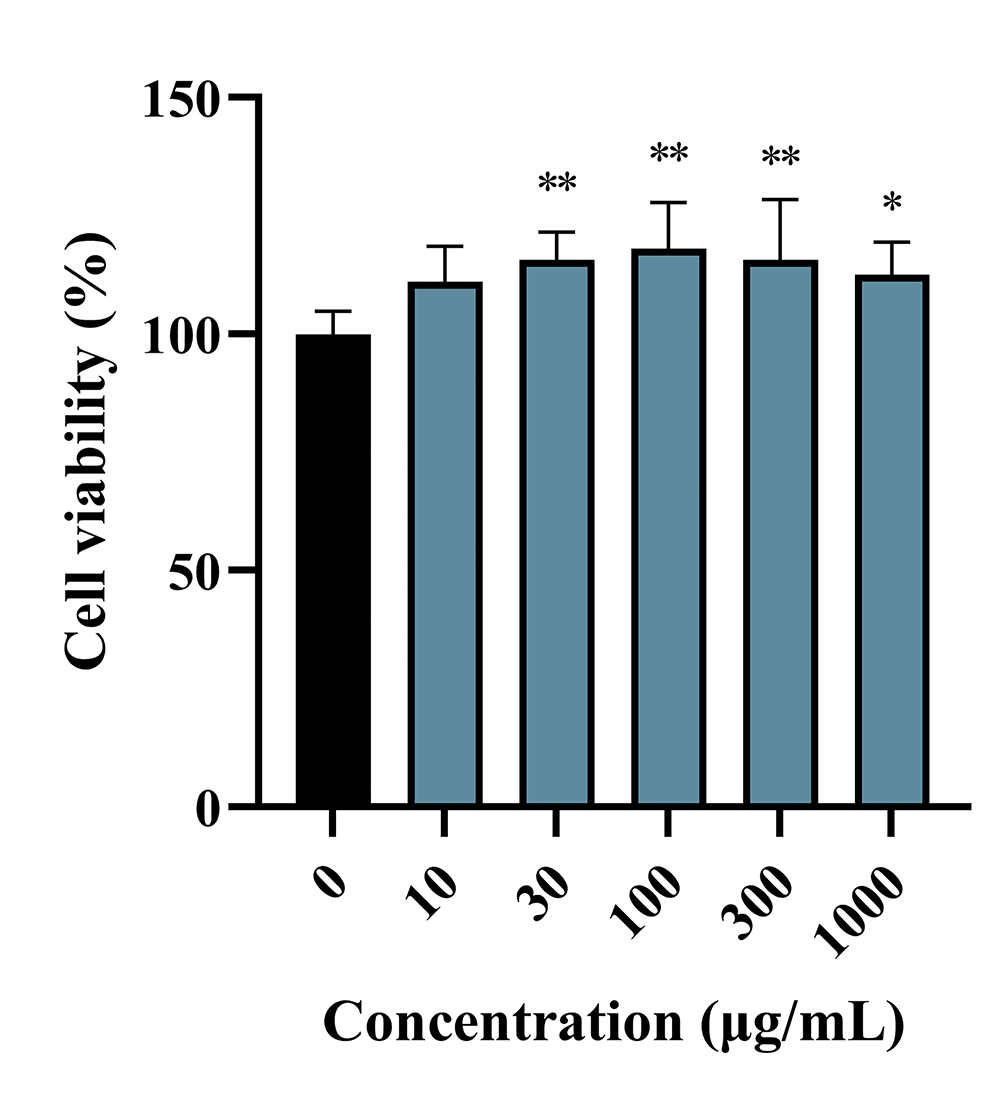
**

**Fig. S2.** Effect of different concentrations of SSP on HaCaT cell viability.
